# The Influence of Markov Decision Process Structure on the Possible Strategic Use of Working Memory and Episodic Memory

**DOI:** 10.1371/journal.pone.0002756

**Published:** 2008-07-23

**Authors:** Eric A. Zilli, Michael E. Hasselmo

**Affiliations:** Center for Memory and Brain, Boston University, Boston, Massachusetts, United States of America; University of Southern California, United States of America

## Abstract

Researchers use a variety of behavioral tasks to analyze the effect of biological manipulations on memory function. This research will benefit from a systematic mathematical method for analyzing memory demands in behavioral tasks. In the framework of reinforcement learning theory, these tasks can be mathematically described as partially-observable Markov decision processes. While a wealth of evidence collected over the past 15 years relates the basal ganglia to the reinforcement learning framework, only recently has much attention been paid to including psychological concepts such as working memory or episodic memory in these models. This paper presents an analysis that provides a quantitative description of memory states sufficient for correct choices at specific decision points. Using information from the mathematical structure of the task descriptions, we derive measures that indicate whether working memory (for one or more cues) or episodic memory can provide strategically useful information to an agent. In particular, the analysis determines which observed states must be maintained in or retrieved from memory to perform these specific tasks. We demonstrate the analysis on three simplified tasks as well as eight more complex memory tasks drawn from the animal and human literature (two alternation tasks, two sequence disambiguation tasks, two non-matching tasks, the 2-back task, and the 1-2-AX task). The results of these analyses agree with results from quantitative simulations of the task reported in previous publications and provide simple indications of the memory demands of the tasks which can require far less computation than a full simulation of the task. This may provide a basis for a quantitative behavioral stoichiometry of memory tasks.

## Introduction

Studies of the biological mechanisms of memory function utilize behavioral tasks that require use of memory systems for successful performance [1]. These behavioral tasks are often designed to test one of many specific hypothesized memory systems [1]–[3]. However, the mechanisms of memory function required for specific tasks often becomes the focus of debate, as there is no quantitative framework for describing the memory demands of individual behavioral tasks. Often memory tasks can be performed by more than one memory mechanism [4]. Even when it is clear what type of memory is required at one point in the task, there might be a different memory requirement at other times, or there might be a need for interaction of different memory systems.

This paper presents mathematical procedures that can be used to evaluate the memory demands at specific decision points in specific memory tasks. The analyses are based around Markov decision processes (MDPs), which provide a framework for describing complex decision processes in behavior [5]. By definition, each decision in a Markov decision process depends only upon the current state [5]. Tasks that require memory can be written as partially-observable Markov decision processes (POMDPs; [6], [7]). Agents can be trained to use memory mechanisms in a non-Markov decision process [4], [8]–[10], but a systematic mathematical process for analyzing the memory demands of a task has not been presented. Analyzing tasks written as POMDPs can help elucidate the memory mechanisms sufficient to solve each memory task, and could provide quantitative details of these memory processes. Analyses examining specific ambiguous observations allow consideration of a range of memory demands within a single task. Thus, analyses of tasks as POMDPs can help provide a quantitative behavioral stoichiometry of memory tasks, providing a solid framework for evaluating potential physiological mechanisms. Using these procedures, behavioral scientists can quantitatively define the type of memory and content of memory sufficient for making decisions at specific points within a behavioral task.

Here we specifically address the use of working memory and episodic memory (and, briefly, time-varying contextual information) for performance of behavioral tasks. These terms have been defined in other research, but are used in a specific, task-independent manner in this paper. Here, working memory refers to active maintenance of information about prior observations, consistent with the use of the term in models and experimental data focused on persistent spiking activity during the delay period of a behavioral task [4], [8]–[14]. Episodic memory refers to storage of a sequence of observations that can be retrieved in response to a single initial cue (the most recent sequence beginning with the cue is retrieved). This operational definition proves useful in behavioral tasks [4], [15], but does not address all the components of the definition of episodic memory in humans [16]. For this reason we refer to this system as content-addressable sequential retrieval (CASR) instead of episodic memory. Finally, our consideration of context deals with systems that provide contextual information as a function of the agent's recent history.

We analyze tasks from the points of view of these memory systems by using what we call disambiguation matrices. These matrices relate observations held in memory at a particular decision point in a task to the possible states the task might be in. When an observation in memory disambiguates a choice point, an agent can learn a policy that more closely reflects the underlying dynamics of the task at that point. By calculating these matrices for different memory systems, we show that tasks can be quantitatively analyzed in terms of which memory systems and which strategies using those systems are useful in performing them.

The essence of these analyses is this: by the structure of the state space of a task as well as by the function of a particular memory system, at any choice point in a task there are only certain observations that can be provided by the memory system. When a choice point in fact corresponds to multiple distinct states (which the agent cannot distinguish between through sensory input alone), it can be that certain observations can only occur in memory when the agent is at certain of the distinct, but superficially indistinguishable states. In such a situation, the memory disambiguates the choice point, providing non-sensory information as to the agent's true state.

For the working memory analyses, we can look backward in time from the choice point to see the recent observations that the agent may have held in working memory. From each of those observations we can look forward to see which choice point states are reachable. If it is only possible for the agent to have observation X in working memory when at choice point state 1, then if the agent has X in working memory, it must be in state 1. In the case of episodic memory, we look as far back in time from the choice point as is needed to find the last time the choice point occurred, then look forward to see, first, what observations can follow that past appearance and, second, which choice points are then reachable from those observations. The following analyses provide a way to answer these questions, using matrices as “bookkeeping” tools to keep track of which observations lead to which states.

The analyses are done from the viewpoint of an omniscient observer who knows the complete description (in terms of a POMDP) of a behavioral task. The results of the analyses are thus primarily useful to those designing or simulating tasks or studying the use of memory systems. The analyses and results from them are less likely to be useful to agents actually performing any particular task, thus the present results are primarily useful as a theoretical tool for understanding and categorizing behavioral tasks.

## Methods

We will be dealing with a type of partially-observable Markov decision process. Let *T* be a POMDP describing the dynamics of a behavioral task. It is a tuple *T* = <*T_S_*, *T_O_*, *T_A_*, *T_P_*, *T_R_*> of, respectively, a set of states, a set of observations, a set of actions, a set of transition probabilities, and a real-valued reward function.

*T_P_* can be written as the set of probabilities *T_P_(s,o,a,s′) = Pr(s_t+1_ = s′, o_t+1_ = o|a_t_ = a, s_t_ = s)* which describes a new state *s_t+1_* and observation *o_t+1_* given a current state *s_t_* and an action *a_t_*. In a POMDP, it is assumed that the underlying dynamics of the environment are Markov but due to hidden variables or limitations in, for example, sensory capability, the agent is not aware of its complete state. Instead it must base its decisions on its current observation.

Our analysis will be restricted to a subset of POMDPs that we call aliased MDPs (AMDPs). An AMDP is simply a POMDP where there exists some aliasing map *A∶T_S_→T_O_* such that that *T_P_(s,o,a,s′)* is only nonzero for *o* = *A(s)* for all *s,a,s′*. That is, the transition probabilities are limited in that only a single observation can occur for any particular state (although many states may be aliased to the same observation). If *A* is one-to-one then the AMDP is an MDP. It is primarily for conceptual simplicity that we make this restriction. The analysis results hold when applied to POMDPs (simply by changing the definition of the aliasing map to be *A∶T_S_→P(T_O_)* where *P(T_O_)* is the set of all subsets of *T_O_*).

This aliasing function is intended to represent the fact that the dynamics of the world can be a function that depends on variables not directly observable by the agent (e.g. in a spatial alternation task, a hidden variable that affects the reward function is the spatial response the agent made on the previous trial). These variables can be included as part of the states *T_S_* so that state transitions and the reward function depend on them, but they may be aliased out so that multiple states in *T_S_* (e.g. with different values of the variables) are treated by the agent as a single observation.

We will often be concerned with the image *A(s)* of a state *s∈T_S_* (i.e. the observation corresponding to state *s*) and the preimage *A^−1^(o)* of an observation *o∈T_S_* (i.e. the set of all states that the agent observes as *o*).

The states *T_S_* will be labeled using the Euclidean basis vectors *e_i_* for *1*≤*i*≤*|T_S_|*. All elements of vector *e_i_* are 0 except for the *i* ^th^ which equals 1. For example, *e_2_ = (0 1 0 0 …)*. We use these so that the states can be directly included in equations of matrices that refer to them. For most of the examples in this manuscript, the observations will be labeled as colors such as green or blue. Observations can also be associated with Euclidean basis vectors, and the aliasing map *A* has a natural extended interpretation as a *|T_O_|*-by-*|T_S_|* matrix mapping the state vectors to observation vectors. This aliasing matrix will be written A.

An important distinction must be made between three similar concepts: state, observation, and what we will call policy-state. States and observations are elements drawn, respectively, from *T_S_* and *T_O_* and are part of the formal definition of a POMDP. We distinguish these from the policy-state of an agent, which refers to the specific information used to select an action when the agent is interacting with the environment (for instance, both the agent's current observation as well as any memory information available to it, [4], [8]–[10]). Policy-states in this manuscript only come into play when considering how the agent can use disambiguation information provided by memory.

Our primary concern in the following analyses are with the topology (or connectivity) of the state space of a task and not with the particular actions that carry the agent from state to state. Because of this, matters are simplified: ignoring the control aspect allows us to write the state space as a Markov chain [17], which we will call *N*. To do so, we construct *N* by assuming the agent always selects an action at random. The following common results on Markov chains will be used.

Row *r* of *N*, *N(r,·)*, is a vector where entry/column *c* is the probability that the agent will move from state *r* to state *c* under one step of its random policy (the probabilities *T_P_(r,o,a,c)* averaged over all *a∈T_A_* and *o∈T_O_*). In Appendix S1 we show that the later analyses give equivalent results regardless of the way *N* is formed as long as each entry is some linear combination with nonzero coefficients of the transition probabilities. This means one could instead calculate *N* as the adjacency matrix of the state space graph, if that is more convenient.

The element at row *r* and column *c* in the matrix *N^n^*, similarly, gives the probability that the agent will be in state *c* after taking *n* random steps from state *r*.

The direction of the transitions in the chain represented in *N* can all be time reversed by transposing *N* (and normalizing the row sums so that they remain probabilities):
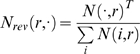
(1)


Row *r* in *N* can be extracted by left multiplying *N* by a unit row vector *e_r_* in which the *r* ^th^ component equals 1 and all others equal 0.

A state is called absorbing if the transitions leading out from it lead only back into itself. State *r* in *N* is absorbing if *N(r,·) = e_r_*. Therefore, the chain *N* can be modified to set state *r* as absorbing by assigning *N(r,·)←e_r_*.

Our results involve disambiguation matrices. A disambiguation matrix *D* here is defined at an observation *o*. The columns of *D* each correspond to a state in *A^−1^(o)*. The rows of *D* each correspond to an observation the agent might have in memory (for instance held in working memory or retrieved in CASR memory). *D* expresses the probability that the agent might be in each state (column) given that a particular observation (row) is in memory. That is, *D(r,c) = Pr(s_t_ = s_c_|o_m_ = o_r_)* where *s_c_* is the state corresponding to column *c*, *o_r_* the observation corresponding to row *r*, *s_t_* the agent's current state, and *o_m_* the observation in memory (reflecting the observation observed at some earlier time). Of particular importance are rows where one or more the probabilities is 0, indicating that, when the row's corresponding observation is in memory, there is less uncertainty as to the current state.

### Tasks

We will derive a simple structural analysis of behavioral tasks in terms of matrices calculated from AMDPs by considering a simplified alternation task. Further examples of the analysis are given for other simplified tasks before we finish by analyzing the tasks exactly as simulated in [4] and discussing the results. We also show how to extend the working memory analysis to the case of holding multiple items in working memory in two additional tasks.

GNU Octave 3.0.0 scripts (MATLAB compatible) containing all of the analyzed AMDPs and analysis functions discussed in this manuscript are available upon request.

#### Alternation

**Figure 1:**
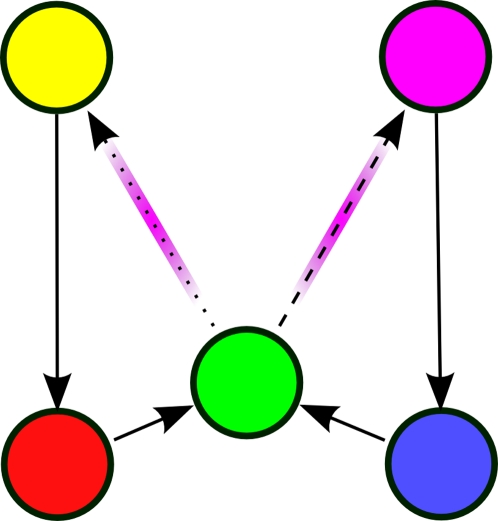
Observed alternation task. This shows the structure of the task as observed by the agent. Transition arrows colored magenta indicate transitions that may provide either positive or negative rewards (depending on whether the agent has alternated or has selected the same response).

From the agent's point of view, our simplified alternation task consists of 5 observations, as shown in Figure 1. This is a greatly abstracted version of the spatial alternation task used in experiments [18]–[20]. A trial consists of the agent passing from either red or blue observations through green to one of yellow or magenta, from which the agent is returned to red or blue, respectively. The agent receives a positive reward for alternately entering the yellow and magenta observations on each visit to green. Because the sign of the reward in going from, e.g., green to yellow does not depend on the agent's observation, but rather on its unobserved state, this chain is not an MDP, but it is expressible as an AMDP.

**Figure 2:**
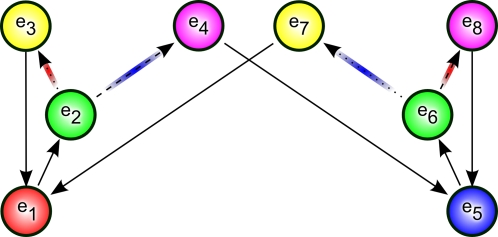
Underlying state-space of the alternation task. The eight states are labeled with vectors *e_1_,…,e_8_*. The five observations are identified by color. Thus there are two unaliased states and three pairs of states that are each aliased to a single observation. Solid arrows indicate transitions that result from any action. Action-specific transitions are indicated by dotted and dashed lines. Red arrows indicate transitions producing negative rewards; blue arrows indicate transitions with positive rewards.

We assume that the underlying AMDP is fully known for the purposes of this analysis. One possible AMDP describing the task with 8 states is shown in Figure 2. States *e_3_* and *e_7_* (respectively *e_4_* and *e_8_*) are distinct only for clarity. The results of this analysis are unchanged if each pair is merged into a single state.

#### Cued Alternation

**Figure 3:**
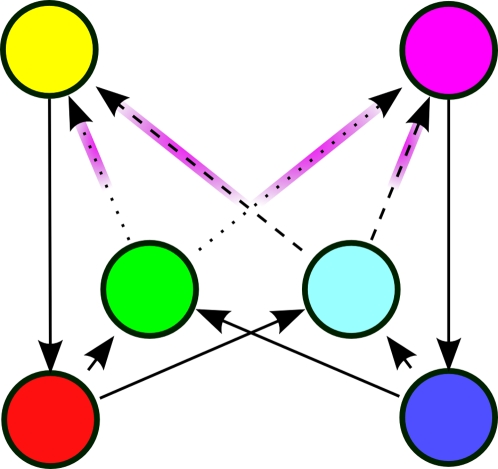
Observed cued alternation task. This shows the structure of the cued alternation task as observed by the agent. Starting at red or blue, one of the two cues green or cyan is randomly selected and the agent can enter either the yellow or magenta observations, before returning to the bottom for another trial. Transition arrows colored magenta indicate transitions that may provide either positive or negative rewards (depending on whether the agent has alternated or has selected the same response for the current cue).

**Figure 4:**
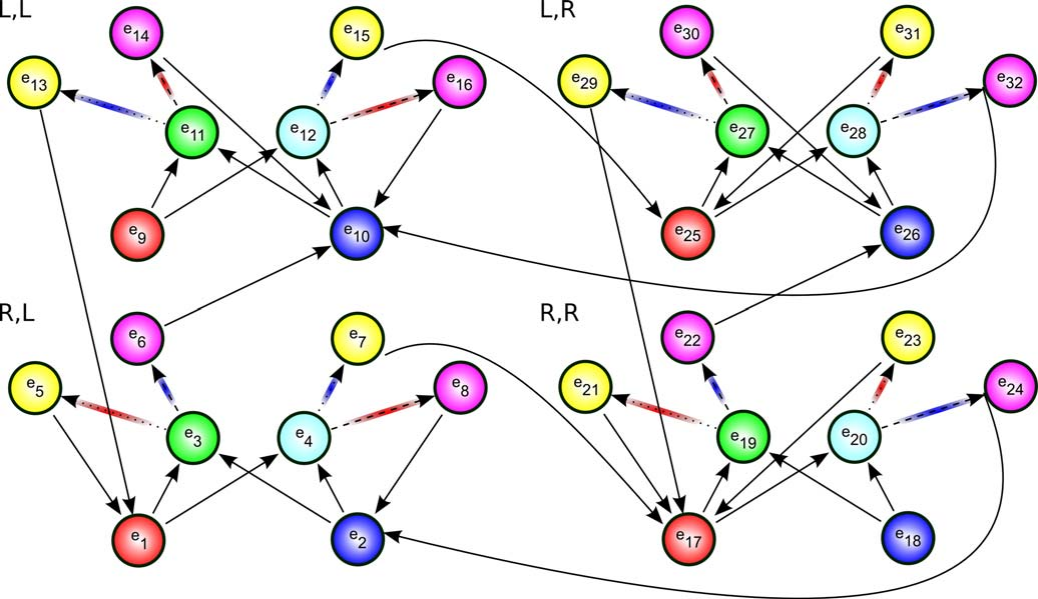
Underlying state-space of the cued alternation task. Each quadrant consists of eight states which differ only in the connections into the red and blue states and out of the yellow and magenta states. The quadrants are identified by letter pairs that correspond to the rewarded actions in each type of trial in the original task. The pair (L,R), for instance, means that if the green or cyan stimulus were presented, the agent would be rewarded for selecting the “L” or “R” action, respectively. Red arrows indicate transitions producing negative rewards; blue arrows indicate transitions with positive rewards.

Cued alternation is a variation on the alternation task. The main difference is that there are two choice points in this task, green and cyan, one of which is selected randomly each trial. The agent is to learn two independent alternations. For instance, each time green is presented, the agent is to alternate its response, regardless of the responses made at any number of intermediate cyan observations. This task is demonstrated graphically in Figures 3 and 4.

#### 2-Back

In the 2-back task (more generally the *n*-back task; [21]–[23]), subjects are given a continuous stream of cues and must respond to a cue only when it matches the cue from two items earlier. The subjects must constantly update their working memory of the most recent cues, because memory of the cue from time *t*-2 is required to respond at time *t*, but memory of the cue from *t*−1 must be maintained in order to respond to the cue at time *t*+1. The version of this task that will be analyzed is somewhat simpler than most versions in that we use only four different cues in the sequence, although the analysis should not differ for a larger set of stimuli.

#### 1-2-AX

The 1-2-AX task ([8], [9]; based on an earlier task from [24]) consists of a stream of the characters {1, 2, A, B, C, X, Y, Z}. For instance, the following stream might occur: 2-A-Z-B-Y-1-C-Z-B-Y-C-X-A-X-…. First a digit 1 or 2 is presented, then two, four, six, or eight letters are presented, one at a time, alternately drawn from the sets {A, B, C} and {X, Y, Z}. In this task, the agent is to make a response when a target sequence appears, where the target sequence depends on whether a 1 or a 2 most recently occurred in the string. If a 1 most recently occurred, the agent should respond to an X if immediately preceded by an A (e.g. the final symbol in the example string above). If the most recent digit was a 2, the agent should respond to a Y preceded by a B (e.g. the fifth symbol in the example above). The probability of a target sequence appearing as a letter pair in the sequence is 0.5, although the results of the analysis are independent of this probability.

In this task, when observing an X or a Y, the agent must recall both which digit was most recently shown as well as the identity of the preceding symbol in order to act optimally.

#### Other Tasks

The six tasks simulated and fully described in [4] will also be analyzed: spatial alternation, tone-cued alternation, spatial sequence disambiguation, odor sequence disambiguation, non-matching to position, and non-matching to lever. The spatial and tone-cued alternation tasks are essentially identical to the simplified tasks described above, differing only in their greater number of ambiguous states and their longer side paths. The spatial sequence disambiguation task is similar to the sequence disambiguation task analyzed in Appendix S2 and involves two sequences of states with overlap in one or more ambiguous observations. In the odor sequence disambiguation task, the agent is presented with pairs of odors it can freely sample (sniff at) before selecting one as a response. There are two sequences of correct odors which overlap in the middle two odors. The choice point is the final pair of odors, where the agent must recall which sequence is being presented. Finally, in the non-match to position and non-match to lever tasks, the agent is first forced to enter one of two positions or press one of two levers. Then both positions or levers are made available and the agent is rewarded for selecting the position or lever that was not available during the first stage.

## Results

### Working Memory Analysis

By working memory [25], [26] we mean the capacity for the agent to hold onto an experienced observation over a number of steps (the definition used in the behavioral simulations in [4], [8]–[10]). In this case, the agent's policy-state at time *t*, *p_t_*, is a triple *p_t_ = (o_t_, o_t−i_, i)* consisting of its current observation *o_t_* and the observation *o_t−i_* that was present when the agent last took its “hold in working memory” action, *i* steps earlier.

An agent or animal that does not have access to the age of a memory (the number of steps it has been held in working memory) may not be able to fully take advantage of the disambiguation information that we will discuss in detail below. This information is not always required, however, as demonstrated by successful working memory simulations that have not included it [4], [8]–[10]. We will return to this in the Discussion section.

#### Example: Alternation

Consider the 8 state, 5 observation alternation task shown in Figures 1 and 2. The states in *T_S_* are labeled with vector identifiers (*e_1_*, *e_2_*, etc.), and color-coded according to their identity in *O* (e.g. *A(e_2_)* = *A(e_6_)* = green). The goal of the task is for the agent to alternate its response on every visit to the choice point (green states). For example, it should always make a “right” response at *e_2_* and a “left” response at *e_6_*.

**Figure 5:**
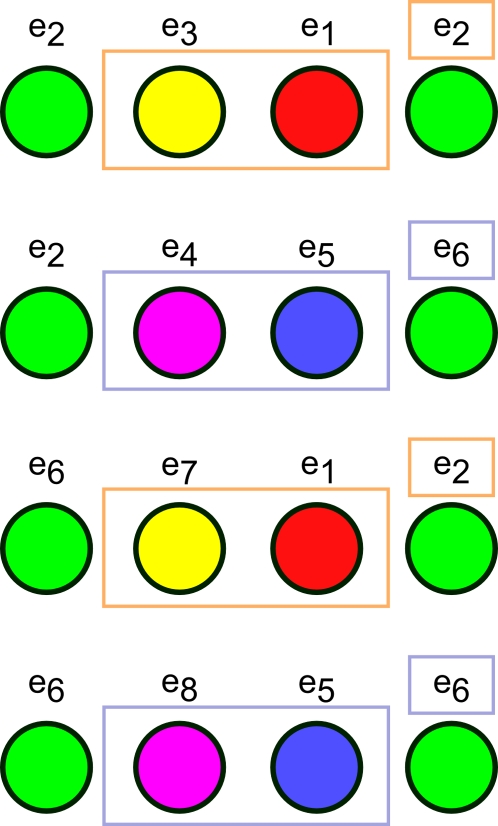
Possible paths in the 5-state alternation task leading from one green state to another. This set of possible paths is the set of possible CASR memories the agent may retrieve from a green cue. In this case, both the second and the third observations that occur in every possible path predict which of *e_2_* or *e_6_* the agent will next enter.

It is clear that the resulting state and reward from taking a given action in state *e_2_* are not the same as those when taking the same action in state *e_6_*. When observing green, the agent cannot learn which is the optimal action to take. However, certain observations in the paths leading into green always predict the agent's current state. If the agent has held its previous observation in working memory, its policy-state will either be (green, red, 1) or (green, blue, 1). For instance, if the policy state is (green, red, 1), then the agent must be at *e_2_*, as is clear in Figure 2. Thus,
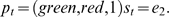



This is made most clear by explicitly listing the paths that can lead into a green state, shown in Figure 5. We see that the agent has policy-states that indirectly come to represent the true underlying states, thus disambiguating an observation. When that happens, the Markov property of a particular aliased state is restored, as just demonstrated. In such a case, working memory allows the agent to learn a policy that more closely relates to the underlying MDP, at least at a single observation.

This result is intuitively clear and, of course, can be determined simply by inspection of the state space for small tasks like the present example. Our goal in what follows is to formalize and automate the process of determining when an observation is disambiguated by working memory and then to extend this to the somewhat more complex case of CASR memory.

In order to concisely express the disambiguation results from above, we write a disambiguation matrix *W* for the observation green. There will be one column in this matrix for each state aliased to green and one row for each observation that may be held in working memory from one step earlier. To specifically indicate that we are considering observations from one step into the past, we refer to the matrix as *W_1_[green]*. We can write the results above as
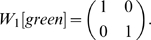



The first column corresponds to *e_2_* and the second to *e_6_*. The first row corresponds to red and the second row to blue. A nonzero entry in the row corresponding to observation *o* and the column corresponding to *s* indicates that the agent can be in state *s* given that *o* is in working memory from one step earlier. If the entry equals zero, the agent cannot be in *s* when *o* is in working memory from the previous step.

We can derive this from the aliasing function *A* and the Markov chain *N* of this task, calculated as described in the Methods (or by inspection of Figure 1). *N* describes the potential transitions between states in the task.
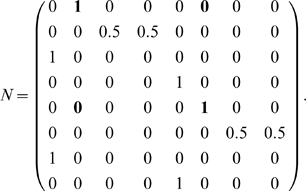



From this we want to calculate *W_1_[o]* for some number of steps *i* and some observation *o*. The columns of *W_1_[o]* correspond to states *A^−1^(o)* (the states aliased to *o*). Each row corresponds to an observation that can occur *i* steps before *o*. Notice that in the present case, this matrix is a submatrix of *N*. Dropping all columns except the second and sixth (for states *e_2_* and *e_6_*), and all rows except *e_1_* (red) and *e_5_* (blue) gives *W_1_[green]* from above, as indicated by the bold elements in the matrix above.

To automate the finding of these observations, we begin with a vector representation of the states aliased to *o*: 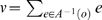, where the nonzero entries correspond to states aliased to *o*. Letting *N_rev_* be the time reverse of *N*, we calculate the new vector 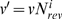, where the nonzero entries correspond to states exactly *i* steps before the states in *A^−1^(o)*.

In the present example, *v = (e_2_+e_6_)*, and
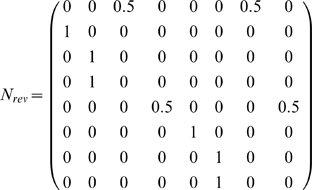
So *v′ = vN_rev_ = (1 0 0 0 1 0 0 0)*. We see that states *e_1_* (red) and *e_5_* (blue) precede green states. Thus the rows of *W_1_[green]* will correspond to red and blue.

We form a matrix 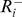 to hold this intermediate computation and to group the states that can occur *i* steps before states *A^−1^(o)* by the observations they are aliased to. We can then left-multiply *N* by 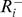 to extract only the rows of interest. In the present example, 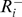 will have two rows: 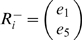. If multiple states in *v′* were aliased to the same observation, the appropriate row in 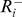 would be the average of the state vectors (see *W_2_[green]* below). We write this formally by using the aliasing matrix A to transform state vectors to their corresponding observation vectors: 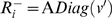 where *Diag(v′)* is a matrix that is all zeros, except along the diagonal where it has the elements of *v′*. Note that the rows of 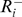 need to be normalized after this step so that they remain probabilities and the rows that are all zeros can be dropped for conciseness.

Similarly, we want to keep only the second and sixth columns. We do so by right-multiplying *N* by a matrix *C* with two columns, 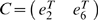.

Together, this yields
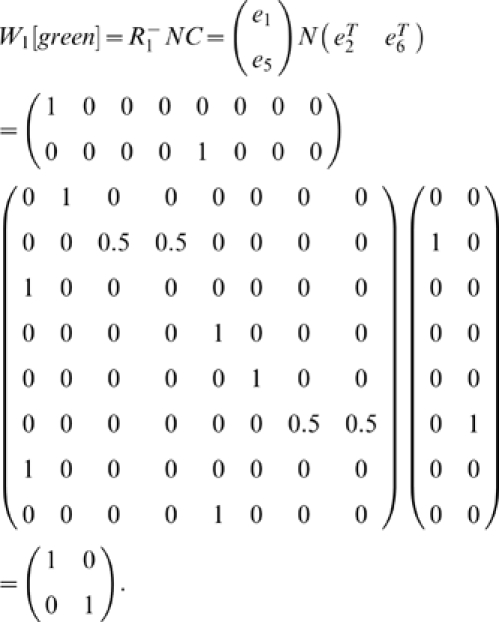



Thus, this mathematical process provides the disambiguation matrix discussed earlier. Notice that this disambiguation is policy independent. By the very structure of the task, working memory can always provide sufficient information to disambiguate the two aliased states, though a policy need not take advantage of this fact.

We can similarly find *W_i_[green]* for other values of *i*:
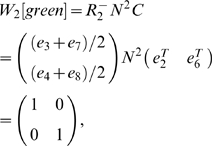
where the rows correspond to yellow and magenta. The rows of 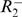 are averages of vectors because, e.g., a yellow observation in working memory might have been either state *e_3_* or *e_7_* (Appendix S1 shows that any linear combination with nonzero coefficients of the vectors produces equivalent results).
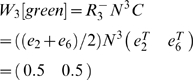



Calculating *W_i_[green]* for *i*>3 shows that elements held in working memory for more than two steps in this task provide no disambiguation.

Consider also 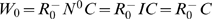. 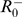 corresponds to a single observation so 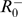 will be a single row. If *C* is a single column (i.e. the state is not aliased), then 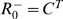 so *W_0_ = 1*. Otherwise *W_0_* is a row vector with *|A^−1^(green)|* elements all equal to *1/|A^−1^(green)|*. For instance,
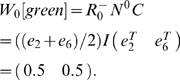



#### Example: Cued Alternation

Next we examine a more complex task. This task, cued alternation, is a simplified version of the task initially described in [4]. The environment has 6 observations (shown in Figure 3) with 32 total states (see Figure 4).

The goal of this task is for the agent to learn two concurrent alternations, alternating separately for green and cyan cues. For convenience we call the two actions “L” and “R”. There are essentially four “trial types” in the task. At any given time, the green cue might require the “L” action (for which there are two possibilities: one where the cyan cue requires the “L” action and another where cyan requires “R”) or green may require the “R” action (for which there are two other possibilities), see Figure 4. We write the possibilities as, for example, (R,L), indicating green requiring “R” and cyan “L”.

For the purposes of this example, we are interested in whether the agent can distinguish between green states requiring “L” vs. “R” responses (a similar analysis is possible for cyan states). Thus the agent, at a green state, should distinguish (L,R) from (R,R), but not from (L,L), which is behaviorally equivalent. We will see how this is taken into account when constructing the matrix *C* below.

The transition matrix for this task is a pair of unwieldy 32-by-32 matrices that are not included here.

For working memory held over one step, we again set 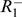 based on the states that transition into green states. As Figure 4 shows, there are four red and four blue states that transition into green. However, states *e_9_* and *e_18_* cannot be occupied except possibly on the first step of a task if the agent begins at them, because they have no incoming transitions. 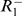 reflects the states the agent may have just occupied, so in general the agent will not have been at *e_9_* or *e_18_* and so they will be omitted. Thus:
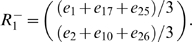



For the analysis of two steps while holding an observation in working memory, we have yellow and magenta rows:
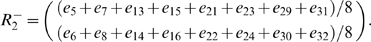



*C* is based on *A^−1^(green)*:
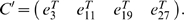
However, recall that green states in (L,R) and (L,L) trials are considered equivalent (disambiguating them is task-irrelevant as mentioned earlier), as are (R,R) and (R,L). Since columns give the probabilities of being in the respective states, we can simply sum columns to lump states together. We use
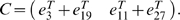



From these we can find
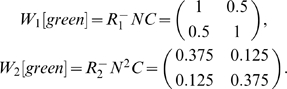



Notice that the rows do not sum to 1 here. This occurs because, unlike in the previous example, green here is not a “bottleneck”; the agent may enter the cyan observation instead of green. Since we are currently only interested in the green states, we can simply normalize the row sums to more easily see the relative probabilities.
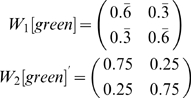



We see that *W_1_[green]* fails to fully disambiguate any state. *W_2_[green]* is halfway between perfect disambiguation and chance level. It is beyond the scope of this analysis to determine in detail what effect on behavior this imperfect disambiguation might produce. However, simulations of this task [4] did show that an agent with only working memory was able to maintain a level of performance intermediate between chance and perfect, correctly responding approximately 2/3 of the time as *W_1_[green]* would predict. This suggests that future work might further elucidate a more general connection between *E_i_*, *W_i_*, and performance level.

*W_1_[green]* for *i*>2 also produce no disambiguation. We will see later that CASR memory, on the other hand, does provide disambiguation in this task.

#### Working Memory Summary

Given a matrix *N* describing the transitions available to the agent and an aliasing function *A∶T_O_→T_S_*, we can ask if the structure of *N* and *A* allows working memory to disambiguate the different states mapping to some particular observation *o∈T_O_*.

The two steps as performed above are: 1. Find matrices 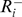 and *C*. 2. Find the product 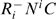.

First, letting 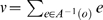, we calculate 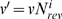 and then 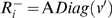, dropping the rows that equal zero and normalizing the other rows. There is one nonzero row in 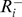 for each observation found *i* steps before the starting states, averaged across each state in the preimage of the observations. There is one column in *C* for each state in *A^−1^(o)*. For simple tasks, 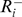 can be determined by inspection of the Markov chain by identifying the states from which *o* is reachable in *i* steps (i.e. it represents the states *i* steps backward from *o*).

Optionally, at this point, the decision should be made regarding which state disambiguations are important and *C* altered appropriately by summing columns. Skipping this step considers all possible distinctions.

Finally, the product 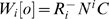 is the disambiguation matrix, which essentially is a submatrix of *N^i^* where certain rows or columns may have been linearly combined.

This submatrix summarizes the chain leading from states in 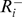, through *i* steps, up to the states aliased to the agent's current state *C*. So, if row *r* and column *c* of the matrix is 0, then the agent cannot arrive at state *c* if the agent's working memory contains *r* from *i* steps earlier.

### Content-Addressable, Sequential Retrieval Memory Analysis

Episodic memory is a form of memory that, in humans, is described as long-lasting and allows a person to recall spescific autobiographical events [16]. Based on an earlier neural network model of the hippocampus in which episodic memories were retrieved on every time step [15], an abstract model of episodic memory has been proposed and simulated in [4]. Here we refer to this as a content-addressable, sequential retrieval (CASR) memory system and assume it is ideal (noiseless and of infinite capacity), containing a copy of the agent's entire history of observations. This CASR memory system allows an agent to retrieve a sequence of observations from its history, beginning with the time at which the agent last visited its current observation (called the retrieval cue; although the analysis can be extended so that any observation can be used as a cue, to do so is outside the scope of the current paper and will be described in a later publication). In practice, this means that an agent can select different actions depending on the path it last followed after its previous visit to a state that looked like its current observation. The agent's policy-state with CASR memory is a triple *p_t_ = (o_t_, e, i)* of its current observation and the observation it currently has in CASR memory (if any), as well as the number of time it has taken its “advance retrieval” action since it last cued CASR retrieval.

An important aspect of this analysis as presented here is that only the agent's current observation can be used as a retrieval cue, as simulated previously [4], [15]. It is straightforward but outside the scope of this manuscript to modify the following analysis so that any observation can be used as a retrieval cue.

**Figure 6:**
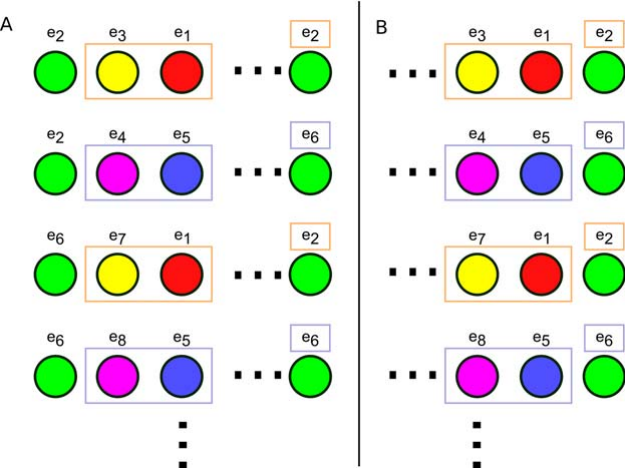
Comparison of the CASR memory and working memory analyses. The possible 5-state alternation paths are shown again, but altered to demonstrate that the paths may be of differing lengths and that there may be many possible paths. A symmetry of the two memory systems is demonstrated by the complimentary way they depend on information from the beginning and end of episodes. A. In the case of CASR memory, observations in each possible episode are examined from the left end of the sequence. These observations correspond to paths out of the observation to be disambiguated. B. With working memory, observations are examined from the right end of the sequence, corresponding to paths leading into the observations to be disambiguated.

In many ways this analysis is similar to that for working memory. There is a symmetry in the two analyses shown in Figure 6.

We begin our consideration of CASR memory with a somewhat general discussion that will use the alternation task as an example (see Figure 2). This will be followed by analysis of one more example tasks (see Appendix S2 in the supporting material for a third worked-out example).

#### Example: Alternation

In Figure 5 are shown all of the possible paths that can take the agent from one green state back to another (possibly the same) green state in the alternation task. These are the possible CASR memories the agent can replay from a green retrieval cue. There are only two unique CASR memories: (green→yellow→red→green) and (green→magenta→blue→green), and each corresponds to two different paths in the state space.

While observing green, suppose the agent cues CASR memory. If the agent takes the “advance retrieval” action once, only a subset of observations in *T_O_* can possibly be retrieved. These are the observations reachable in one step by actions leading out of states *A^−1^(green) = {e_2_, e_6_}*, which correspond to the non-zero entries in the vector resulting from the product *(e_2_+e_6_)N* (i.e. the sum of the second and sixth rows of *N*). As clear from Figure 2 or from *N* itself, this yields the four states {*e_3_*, *e_4_*, *e_7_*, *e_8_*}, aliased to observations yellow and magenta.

We may ask if having experienced any one of these observations forces the agent's state to be either *e_2_* or *e_6_* on its next visit to a green state. More generally, let the agent be observing *o∈T_O_*. By the structure of *T*, does knowing that the previous episode began with the sequence *o→o_a_→o_b_…* provide information as to which state the agent might be in?

An easy way of determining this is to make a new Markov chain *N_abs_* with both *e_2_* and *e_6_* (more generally, states *A^−1^(o)*) set as absorbing states and see if one, both, or neither of these two absorbing states are reachable from each state in the chain.
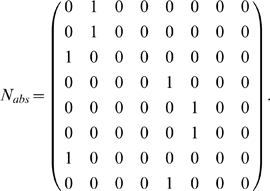



From this, we can use a straightforward tool in Markov chain theory, the absorption probability matrix *B* [17]. *B* is the matrix where *B(r,c)* is the probability that the chain will absorb in *c* when starting from state *r*. To find *B*, the absorbing rows of *N_abs_* are first discarded. The columns of the remaining rows are divided up into one matrix *Q* of columns corresponding to transient states and one matrix *R* of the absorbing state columns (not to be confused with the unrelated matrices 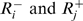 we use elsewhere). Then:
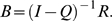
(2)


This is the absorption probabilities only for transient states. For our definition, the matrix *B* must then have the absorbing rows that were removed put back in place (restricted only to the columns used) so that all states are included.
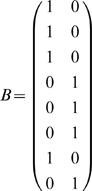



Starting from each state, we see that the agent will eventually be found at either *e_2_* or *e_6_* and that this is deterministic in that, e.g., starting from one of the states *{e_1_, e_2_, e_3_, e_7_}* (rows 1–3 and 7 in *B*) will always result in the agent passing through *e_2_* before *e_6_*. Likewise, starting from one of *{e_4_, e_5_, e_6_, e_8_}* will result in the agent passing through *e_6_* before *e_2_*. Compare this result with Figure 5.

This nearly answers our question about the use of CASR memory in this task. The agent observes that it is in green and through its CASR memory actions can retrieve the prior episode which, let us say, begins *green→yellow→…* The states aliased to yellow that are immediately reachable from green are *{e_3_, e_7_}*. From *B* we see that *e_2_* is the first state in *A^−1^(green)* reachable from both *e_3_* and *e_7_*. Thus: if the most recent pass through green was followed by yellow, the agent must currently be at *e_2_* and cannot be at *e_6_*. The opposite results follow if the prior episode began *green→magenta→…*, in which case the agent must be in *e_6_* and not *e_2_*. We see that CASR memory has fully disambiguated green.

As in the working memory analysis, this result can be made clearer by examining only a submatrix of *B*, removing the information that is not of interest. Instead of calculating 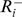 using the states *i* steps before *o*, we calculate 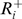 with the states *i* steps after *o*. This is done by finding *v′ = vN* instead of *v′ = vN_rev_*. Proceeding as before, we calculate the disambiguation matrix *E_1_[green]*:
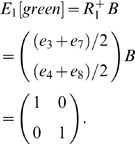



The first and second rows correspond to yellow and magenta, respectively, and the columns correspond to states *e_2_* and *e_6_*.

When calculating 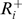 for *i*>1, a slight modification to the above is appropriate. The observations used in making 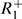 were based on the nonzero entries in *vN*, where *v* was the sum of the state vectors aliased to the agent's current observation. Though one might expect that 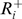 would be based on *vN^i^*, in the present task this results in a repeating sequence of matrices 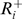. In this alternation task, the nonzero entries would correspond to the sequence of observations {yellow, magenta} for 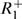, {red, blue} for 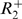, {green} for 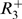, back to {yellow, magenta} for 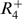, and so forth, repeating forever. However, when CASR memory is cued by a green observation, the retrieved memory begins at the last visit to a green state and can continue only as far as the agent's subsequent visit to green (the present time). So, although *vN^i^* gives the state occupancy probabilities after *i* steps, there may be states *i* steps away that are not actually retrievable. To prevent these from appearing in *v′*, *vN* is used to take the first step out of the retrieval cue states, but the remaining *i*−1 steps are taken in the chain *N_abs_*, preventing retrieval past the current time. Combining these gives 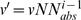.

#### Example: Cued Alternation

We return again to the cued alternation task. Let us consider what information one step of CASR memory provides when used from a green state. We first find matrices *R* and *C*.


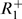
 here comes, as before, from the states immediately reachable from green states. Examining Figure 4 easily provides the information: there are four such yellow states and four such magenta states.
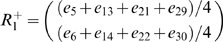



Here we will modify the matrix *C* used in the working memory analysis of cued alternation (because *B* has fewer columns than *N*, but we still want to combine the cue states into two columns):
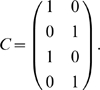



As in the previous example, we set the green states absorbing to find *N_abs_* from *N*, and then calculate *E_1_[green]*.
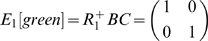



The rows observations are yellow and magenta, and the first column corresponds to states *e_3_* and *e_11_*, while the second column corresponds to states *e_19_* and *e_27_*.

If we had not combined states in making *C*, we would have found
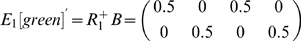
which expresses the same disambiguation information as *E_1_[green]*, but in a less clear manner.

In this task we see that CASR memory for a green cue can disambiguate the agent's state along the green-relevant dimension, and the cyan cue can disambiguate the cyan-relevant dimension (which the reader may verify). So although perfect disambiguation is impossible (as demonstrated by *E′*), the disambiguation is sufficient for performing the task. It is simple to show that for cyan cues, the second letter in the pair can be disambiguated, but the first letter cannot. For example, (R,L) and (L,L) cannot be disambiguated, but they can be distinguished from (R,R) and (L,R).

The same disambiguation occurs in *E_2_[green]*.

#### CASR Memory Summary

Given a matrix *N* describing the transitions available to the agent and an aliasing function *A∶T_O_→T_S_*, we can ask if the structure of *N* and *A* allows CASR memory to disambiguate the different states mapping to some particular observation *o∈T_O_*.

The three steps as performed above are: 1. Find 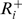. 2. Calculate *B*. 3. Find the product 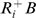.

Letting 
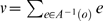
, we calculate 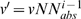, where *N_abs_* has states *A^−1^(o)* set as absorbing. Then 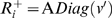. Each nonzero row in 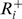 corresponds to an observation found *i* steps from *A^−1^(o)*, and the value of each row (after normalization) is the average of the state vectors aliased to the corresponding observation.

Next we find *B*. Using *N_abs_* from the previous step, *B* is calculated using Equation (2) to identify which of the *o* states the agent will visit first when starting at each state in *T_S_*.

Finally, the product 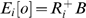 is the disambiguation matrix. *E_i_[o]* summarizes the chain leading from states in 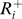, through arbitrarily many states, up to the states aliased to the agent's current state *o*.

The examples used above had a small number of potential episodes that could easily be drawn. However, there could be infinitely many potential episodes and the results would still hold as long as some *i* ^th^ observation always disambiguates the cue state (for an ideal CASR memory with infinite capacity), as suggested by Figure 6A.

This analysis can be extended to allow an arbitrary observation to be used as a retrieval cue. This results in a slightly different interpretation of the disambiguation matrix and introduces other small complexities that depend on the way in which the retrieval cue is selected. Because all of the tasks considered in this paper can be solved using only the agent's current observation as a retrieval cue, we do not consider this modification any further, but leave it for a future paper to examine in more detail.

### Full Tasks

The analysis was performed on simplified tasks above to provide short examples, but it can also be performed on larger AMDPs. We used the algorithm described in Appendix S3 to form AMDPs of the tasks that were simulated in [4]: spatial alternation, tone-cued spatial alternation, spatial sequence disambiguation, odor sequence disambiguation, non-match to position, and non-match to lever.

The implementation of spatial sequence disambiguation contained 18 states aliased to 11 observations. The observations were the agent's spatial coordinates; the states were its coordinates along with an indication of whether the agent should respond by going left or right. The observation of interest here was the choice point at coordinates “2,2”. At this state, matrices *E_3_[2,2]* and *E_4_[2.2]* were identity matrices, as were *W_3_[2,2]* and *W_4_[2,2]*. These matrices reflected the two states on the starting arm of this task: either having recently come from one or the other starting arm (with working memory) or memory of having recently entered one or the other starting arm (with CASR memory).

Our implementation of the spatial alternation task [18]–[20] contained 18 states aliased to 13 observations. The observations were the agent's spatial coordinates; the states were its coordinates along with an indication of whether the agent last went left or right. The observation of interest here was the choice point at coordinates “2,2”. At this state, the matrices *E_1_[2,2]* through *E_5_[2,2]* were identity matrices, as were matrices *W_3_[2,2]* through *W_7_[2,2]*. These both correspond to the 5 state long side arms of the maze. Memory of either having recently been in one (working memory) or recently entered one (CASR memory) and not in the corresponding state on the other arm provide information that allows the agent to perform alternation.

Our implementation of the cued alternation task contained 72 states aliased to 14 observations. The observations were the agent's spatial coordinates and also, only at the choice point “2,2”, one of two cues, selected at random (thus there are actually two choice point states: one for each cue). The states were the agent's spatial coordinates, the most recent tone to have played, and the direction the agent should go on the subsequent presentation of each tone (for 5 state elements in total). The matrices *E_1_[2,2,cue1]* through *E_5_[2,2,cue,1]* were identity matrices (after summing appropriate columns, as in the earlier example). For no *i* was *W_i_[2,2,cue1]* was an identity matrix. Agreeing with the earlier example analysis, *W_3_[2,2,cue1]* through *W_5_[2,2,cue1]* were halfway between identity and chance level.

The non-match to position task [27] comprised 28 states and 10 observations. States were made up of spatial coordinates and an indication of both the current task stage (sample versus test) and which response the agent should make at the choice point. The observations were spatial coordinates, except at the choice point “3,2” where the directions the agent could go were also observed (corresponding to one arm of the maze being blocked, forcing the agent to go the other way, or neither being blocked). The observation of interest was the choice point during the test stage of the task. In this case, the identity matrices were *E_6_[3,2,LR]* through *E_8_[3,2,LR]* and *W_4_[3,2,LR]* through *W_6_[3,2,LR]* (LR meaning that neither possible direction was blocked). These correspond to memory of the choice point and the reward arms during the sample stage.

The non-match to lever task [28] was somewhat more complex than the previous three tasks. This task is made up of 30 states and 18 observations. The observation of interest is one of the two lever states. In the above tasks, the agent was constrained to keep moving forward and so the agent could not re-enter a state immediately after leaving it. In the non-match to lever task, the environment was rectangular so the agent could re-enter a state just after leaving it. Thus there are many more states that can immediately precede or follow a given state. Consider matrix *W_3_[lever1]* for this case (with row sums normalized):
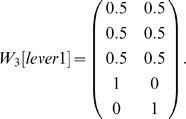



Of the five states that can occur three steps before a choice point, three do not disambiguate (three locations in the space around the levers in the test stage), but two do disambiguate (the two levers from the sample stage). For larger values of *i*, *W_i_[lever1]* shows that, initially, some states continue to disambiguate the observation of interest, but the disambiguation decreases to chance over time. With CASR memory, only partial disambiguation is possible, starting from *W_4_[lever1]*:
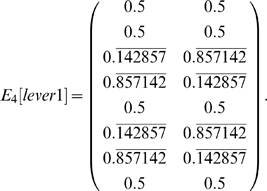



The most complex task in [4] was odor sequence disambiguation [29], which comprised 106 states aliased to 42 observations. In this task, the observations included information as to which of the 5 pairs of odors in a trial the agent was currently at, which odor it was currently smelling, as well as its status as to whether the agent was currently successfully or unsuccessfully attempting to respond to one of the two odors (four such possibilities) or whether it is not currently attempting to responding (a fifth possibility). Observations of interest occur in the final pair of odors (e.g. observation “at pair 5, not responding to an odor, not sampling an odor” or (5,0,0)), where the agent must recall which of the two odor sequences is currently being presented. Here, disambiguation is first provided by matrices *E_5_[5,0,0]* and *W_7_[5,0,0]*. One example matrix is shown below.
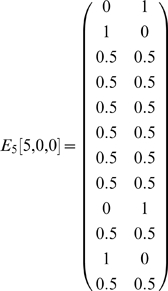



Of the twelve observations that can occur five steps after (5,0,0), four of them provide disambiguation. These four observations are the possibilities of successfully or unsuccessfully digging in one of the two scented cups.

These results are briefly summarized in Table 1.

**Table 1:** Disambiguation results for all analyzed tasks.

Task	Working memory disambiguation *W_i_*[*choice point*]	CASR memory disambiguation *E_j_*[*choice point*]
Simplified Alternation	full (*i* = 1,2)	full (*j* = 1,2)
Spatial Alternation	full (3≤*i*≤7)	full (1≤*j*≤5)
Simplified Cued Alternation	—	full (*j* = 1,2)
Cued Spatial Alternation	—	full (1≤*j*≤5)
Non-Match to Position	full (4≤*i*≤6)	full (6≤*j*≤8)
Non-Match to Lever	full (*i* = 3), decreasing for *i*>3	—
Spatial Sequence Disambiguation	full (*i* = 3,4)	full (*j* = 3,4)
Simplified Sequence Disambiguation	full (*i* = 1)	full (*j* = 2)
Odor Sequence Disambiguation	full (*i* = 7), decreasing for *i*>7	full (*j* = 5), decreasing for *j*>5
2-Back	semi (1≤*i*≤3)	semi (*j*>1)
1-2-AX	semi (*i* = {1,2,4,6})	—

Full disambiguation (e.g. an identity matrix) implies that an agent or animal using the given memory system should be able to perform the task perfectly. The performance of an agent at a state that is semi-disambiguated (defined as at least some zero entries appearing in a nonzero row in the matrix) should be suboptimal or even very poor, as the agent will not have sufficient information to make the correct decisions at the choice points.

### Working Memory of Multiple Observations

The CASR memory and working memory analyses above can be used as a basis for analyses considering more complex combinations and strategies using memory systems. We present one example: an analysis of the case where more than one item must be held in working memory to fully disambiguate an observation. This is motivated by human working memory tasks, which are often more complex in requiring that a subject hold more than one item in working memory at a time [30].

In both the 2-back and the 1-2-AX task, disambiguation of an observation depends on simultaneous working memory of items from multiple time points in the past, e.g. times *t−i*, *t−j*, … We desire a generalization of the earlier analysis to form a matrix *W_i,j,…_* reflecting this multi-item disambiguation. For instance, in the 2-back task where items from both of the two previous time steps we expect that matrix *W_1,2_* should provide complete disambiguation.

Whereas each row of *W_i_* corresponded to a single observation from which the observation of interest is reachable in *i* steps, each row of *W_i,j_* corresponds to a pair of observations, one found *i* steps before the observation of interest and the other found *j* steps before. Thus if there are *r_i_* rows in *W_i_* and *r_j_* rows in *W_j_*, there are *r_i_r_j_* rows in *W_i,j_*. However, *W_i,j_* still has the same number of columns as *W_i_* and *W_j_*.

For convenience, let *O_i_(r_a_)* be the observation corresponding to row *r_a_* in *W_i_* and *S_i_(c)* be the state corresponding to column *c* in *W_i_*. Consider the entry *W_i_(r_a_,c)*. If this entry is zero, then memory of observation *O_i_(r_a_)* held over *i* steps means the agent cannot possibly be in state *S_i_(c)*. If the product *W_i_(r_a_,c)W_j_(r_b_,c) = 0*, then from one or both of the matrices we know that the agent cannot be in state *S_i_(c)*. We can form a row vector of these products for each column by taking the Hadamard (element-wise) product of rows *W_i_*(*r_a_*,•) and *W_j_*(*r_b_*,•).

We define *W_i,j,k,…_*, *i≤j≤k≤…*, to be the matrix composed of all such row vectors (the Hadamard products of one row vector taken from each of *W_i_*, *W_j_*, …). This is the Khatri-Rao product of matrices *W_i_,W_j_,…* with each column as a separate partition, written W_i,j_ = W_i_ * W_j_ * …, and can be formally defined as the partition-wise Kronecker product of *W_i_* and *W_j_* [31], [32].

This gives us the final form for the disambiguation matrix representing the holding of multiple items in working memory:
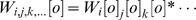
(3)


#### Example: 2-Back

The 2-back task consists of 4 observations (cues) and 36 states (AMDP generated from a simulation of the task per Appendix S3). Each state is an ordered triple of the current and past two cues, e.g. (A,B,C) if cue A was followed by cue B and then cue C. When generating sequences, a given cue can not occur twice in a row, so for any one of the 4 observations, there are 3 observations that can precede it and 3 possible observations that can occur two steps previously, for 36 possible states. States are aliased so that only the final element in the list (the current cue) is observed, so the preimage of each observation contains 9 states. It is these states which must be disambiguated from each other. Without loss of generality, we can select cue A as the observation of interest.

Let us consider single-item working memory matrices first.
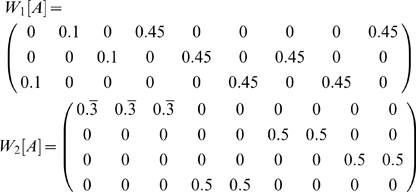



The rows in *W_2_[A]* correspond, respectively, to cues A, B, C, and D. The rows in *W_1_[A]* correspond to cues B through D. The first three of the nine columns correspond to the matching conditions (when an A was presented two steps earlier). The last six columns correspond to the non-matching conditions.

Although both *W_1_[A]* and *W_2_[A]* provide partial disambiguation, none of the nine columns are fully disambiguated. Consider, however, *W_1,2_[A]*.
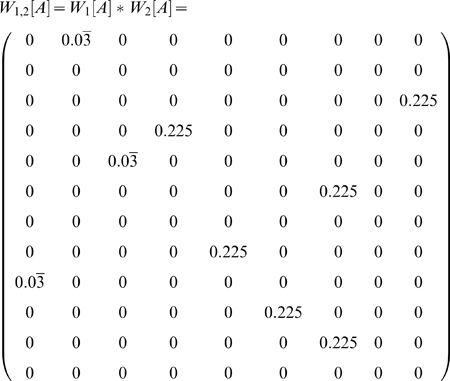



The first row is the Hadamard product of the first rows of *W_1_[A]* and *W_2_[A]*. The second row is the Hadamard product of the first row of *W_1_[A]* and the second row of *W_2_[A]*, and so forth.

Now each state is completely disambiguated. In this case, examining the values of *W_i,j_[A]* for various *i*, *j* suggests that it is only *W_1,2_[A]* that provides perfect disambiguation.

Notice also that there are three rows that equal the 0 vector. These correspond to memories that never occur in the task (recalling the same cue from both 1 and 2 steps into the past).

#### Example: 1-2-AX

The 1-2-AX task comprises 8 possible observations (1, 2, A, B, C, X, Y, Z) and 38 states (AMDP generated from a simulation of the task per Appendix S3). The states include the current observation, an indication of whether the most recent digit was 1 or 2, and the identity of which of the 9 letter pairs is being presented (i.e. if an X is presented, the possible letter pairs are (A,X), (B,X), and (C,X)).

Again, let us begin by examining a few working memory disambiguation matrices. Our observation of interest will be the letter X.
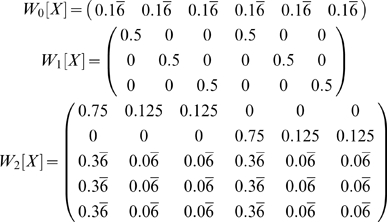



The rows in *W_1_* correspond to A, B, and C (the observations that can precede X). The rows in *W_2_* correspond, respectively, to 1, 2, X, Y, and Z. The first three columns in both matrices correspond to sequences beginning with a 1, the final three columns correspond to sequences starting with 2. The first and fourth columns correspond to an A immediately preceding an X, the second and fifth correspond to a B preceding an X, and the third and sixth to a C preceding an X.
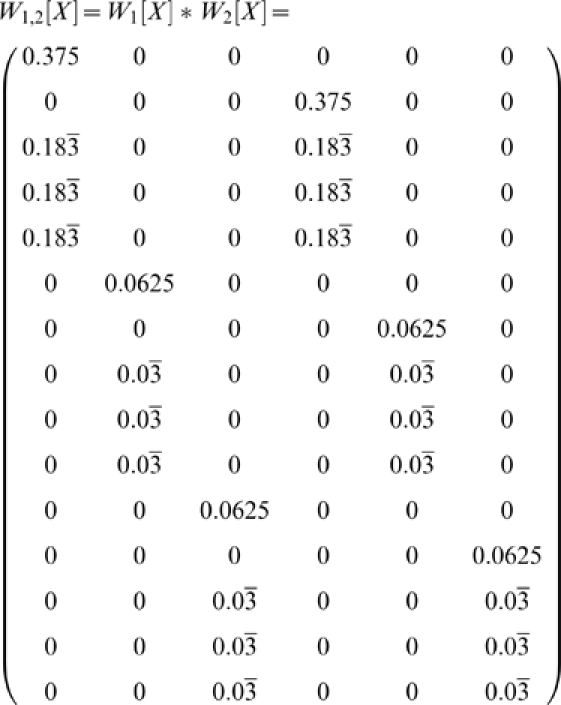



The first row corresponds to an A preceding an X (from *W_1_[X]*) and a 1 preceding the X by two steps (from *W_2_[X]*) which, combined give the sequence 1-A-X. The second row corresponds to the sequence 2-A-X. The sixth, seventh, tenth, and eleventh rows correspond to the sequences 1-B-X, 2-B-X, 1-C-X, and 1-D-X.

Here each column is disambiguated, although there are many rows that do not perfectly disambiguate the states. Unlike in the 2-back task, however, in this task *W_1,2_[X]* is not the only matrix that can disambiguate the states. Similar disambiguation results from *W_1,4_[X]* and *W_1,6_[X]*. An informative digit can also occur eight steps before an X, so *W_1,8_[X]* should provide complete disambiguation. However, consider the valid sequence 1AX2BY2AX. The 1 is eight steps before the final X, but is from a previous trial: eight is the first distance at which a digit from an earlier sequence can be hit, occurring when 2 sequences in a row have only a single pair of letters. Though the agent may still be able to solve the task, the structure of the task does not guarantee that the final letter in a nine item sequence can be disambiguated. Also, perfect disambiguation is never possible when *i*>2 because memory of the immediately preceding stimulus is needed in this task.

### Time-Varying Context

On a disambiguation level, brain systems that provide an ongoing, time-varying context as a function of the agent's experienced observations are related to working memory for multiple items. Formally we define an order-*n* time-varying context as a function *c∶H_n_→T_O_* where *H_n_* is the sequence of the *n* most recently experienced observations. Thus *c* is some function mapping the recent history of the agent onto an observation representing some form sof context. Examples of this type of system include the temporal context model [33] or queue-like buffers [34]. We will consider policy-states of the form *(current observations, contextual observation)*.

At most there can be as many different contexts as there are *n*-observation sequences (in practice, only in an environment where any observation can follow any other would all theoretical sequences actually be possible). In this case, each context would uniquely identify (“sum up”) the current history. So the disambiguation of the context would be 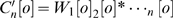 (in fact, this is a conservative calculation, but further details are outside the scope of this discussion and will be provided in a subsequent paper).

It is much more likely that certain sets of histories would produce the same contextual observation, i.e. *c(H_1_) = c(H_2_)* for two histories *H_1_≠N_2_*. The context function acts as an aliasing map on the history of the agent: it combines rows of 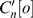 in the same way that the matrices 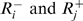 combine rows in *W_i_[o]* and *E_j_[o]*, respectively. Representing the aliasing given by function c in a matrix *C_c_* lets us write the disambiguation matrix 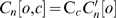.

It is clear that time-varying contextual information can provide disambiguation of an observation. It is important to emphasize, though, that not every memory system can do so. The following section gives an example of a form of memory that never disambiguates observations.

## Discussion

We have demonstrated a simple process for calculating matrices that reveal structural information about a given AMDP and are derived to represent and evaluate the memory demands of a wide range of behavioral tasks. The results of our analyses are summarized in Tables 1 and 2. These analyses may prove useful for evaluating the effect of lesions of brain regions on specific memory mechanisms [20], [26], [36], and for evaluating how patterns of neural activity could mediate different mechanisms of memory function [34]. For instance, the simulation results reported in [4] agreed with lesion studies and the present analyses support those simulation results.

**Table 2:** Disambiguation results in multi-item working memory tasks.

Task	Working memory disambiguation *W_i,j_*[*choice point*]
2-Back	full (*i* = 1, *j* = 2)
1-2-AX	full (*i* = 1, *j*∈{2,4,6}),
	semi (*i* = 1, *j* = 8)

Full disambiguation means that an agent or animal using the given memory system should be able to perform the task perfectly. At a semi-disambiguated state, the performance of an agent may be suboptimal.

It is important to emphasize that the analyses concern the disambiguation of single observations. Whereas it may be common to refer to some task as, e.g., a working memory task or as an operant conditioning task, it is only at particular observations where memory systems are important for making decisions. Consider that the entire life of a laboratory animal may be considered as a single “task”, but it would not be right to consider this task to be a working memory task, or an episodic memory task, or an operant conditioning task, etc. Nevertheless, for tasks that have only a single choice point (there are many such tasks), the entire task could be classified simply according to the strategies useful at the choice point. In this sense, for instance, spatial alternation can be considered both a working memory task and an episodic memory task. Further, tasks with multiple choice points where all the choice points have the same set of useful strategies (e.g. the cued alternation task considered here) can be similarly classified according to those strategies.

All of the tasks analyzed in this paper except the 2-back task have been simulated in previous publications [4], [9], showing that agents can indeed learn tasks that our analysis predicts they should be able to learn. Unpublished simulations of our own show that the 2-back task can also be learned in a manner similar to the methods used in [4].

Although this manuscript has focused on the underlying structure of tasks, the analysis does suggest a prediction regarding neural activity of animals performing episodic memory or working memory tasks. While disambiguation comes in part from the identity of observations in working memory or CASR memory, additional disambiguation information is provided by the age of an item in working memory or the number of steps of CASR memory retrieved. An immediate prediction of this is that there should be a neural representation of this information in addition to a representation of stimulus identity. Successful past simulations of working memory [4], [8]–[10] have not included this information. These simulations were successful because the task were solvable without specifically requiring discrimination based on the age of an item in working memory (though it is straightforward to construct “pathological” AMDPs where such information should be required). However, decisions in the 2-back task do depend upon the age of an item, so this task should show neural activity corresponding to the age of the memory. Responding on the basis of the order of stimulus presentation [35], [36] also requires discrimination of the age of items in working memory. Physiological data suggests that this discrimination of age may be provided by a gradual change in neural activity corresponding to temporal context [33], [35].

In addition to the analysis of existing tasks, these and similar analyses may be useful in the design and evaluation of new tasks. This is a direction we have not yet thoroughly explored, but two possible approaches are clear. First, one might design a task using whatever methods one prefers, then subject the task to these types of analysis, and finally revise the task based on the results (possibly applying multiple iterations). To design a task with specific memory requirements, one might start by writing a set of disambiguation matrices which one desires that an as-yet-unknown task will reflect. These would provide a sort of task skeleton, describing which observations lead to which states through some specific number of steps. While multiple tasks may have the same disambiguation matrices, this approach provides constraints on connectivity which may reduce the complexity of task design.

The present work only begins to consider the full disambiguation problem. This work will be expanded in the future to address additional tasks that require an interaction of a number of memory systems (e.g. the multiple-item working memory example given earlier). Our CASR memory analysis could also be extended to consider CASR memory for sequences of observation-action pairs instead of simply sequences of observations. By taking into account the specific actions taken by the agent, it is likely that additional disambiguation would be provided. Additionally, other memory systems could be analyzed in this framework in the future, for instance, more complex learned context systems [37].

These analyses have used a set of simple techniques which may be useful in analyzing additional brain systems, even those unrelated to the disambiguation problem. Many systems can be translated into the framework of POMDPs and reinforcement learning. Procedural memory might be defined simply as an automatic encoding and playback of sequences of learned actions. Selective attention in the context of factored observations could be treated as the ability to select an action depending on only a subset of the elements in the agent's current observation (those elements that are attended to) using additional actions to selectively attend to or ignore elements of an observation. Sensory memory and priming could be considered as altering the aliasing function from the underlying states of a task (raw sensory input) to the observations on which the agent acts. Theoretical results at even this abstract level will likely be useful in designing new behavioral tasks to study memory, in understanding how neural activity may relate to different strategies that agents or animals can use, and in designing increasingly physiological models of these systems.

## Supporting Information

Appendix S1 (0.06 MB PDF)

Appendix S2 (0.66 MB PDF)

Appendix S3 (0.03 MB PDF)
