# Supplementary material for: The Influence of Markov Decision Process Structure on the Possible Strategic Use of Working Memory and Episodic Memory
Source: PLoS One. 2008 Jul 23;3(7):e2756. doi: 10.1371/journal.pone.0002756 (PMC2447173; doi:10.1371/journal.pone.0002756)
Supplement: Appendix S1 — (0.06 MB PDF) [file pone.0002756.s001.pdf]

The Influence of Markov Decision Process Structure on the Possible Strategic Use of Working Memory and Episodic Memory
Appendix S1

Eric A. Zilli* and Michael E. Hasselmo

* Corresponding author. E-mail: zilli@bu.edu.

Center for Memory and Brain

2 Cummington St.

Boston University

Boston MA, 02215 USA

Ultimately, the results of these analyses are disambiguation matrices where the important information is whether a given entry is zero or nonzero. Of particular interest are rows with a single nonzero entry. Because the values of these nonzero entries are not important, there can be many different m-by-n matrices that contain the same qualitative disambiguation information. We can say two nonnegative matrices X and Y are equivalent, written $X \sim Y$, if $X(r,c) > 0 \iff Y(r,c) > 0$ for all r and c and if the row and column labels (i.e. the corresponding states and observations) of the matrices agree. We define an analogous relation on vectors. This equivalence relation captures the information that is most relevant for the question of the disambiguation of states.

Theorem 1. $X_1 \sim X_2, Y_1 \sim Y_2 \implies iX_1Y_1 \sim jX_2Y_2$ for $i, j > 0$

Proof. The value at row r and column c of iX_1Y_1 is given by $i\sum_k X_1(r,k)Y_1(k,c)$, which is only zero if one or both of $X_1(r,k), Y_1(k,c) = 0$ for all k (because these are nonnegative matrices). But because $X_1 \sim X_2, Y_1 \sim Y_2$, one or both of $X_2(r,k), Y_2(k,c) = 0$ for all k and so $iX_1Y_1 \sim jX_2Y_2$.

Corollary 1.1. $X_1^n \sim X_2^n$. By induction, since $X_1 \sim X_2$, if $X_1^{n-1} \sim X_2^{n-1}$ then $X_1^{n-1}X_1 \sim X_2^{n-1}X_2$.

Theorem 2. $X_1 \sim X_2, Y_1 \sim Y_2 \implies (iX_1 + jY_1) \sim (kX_2 + lY_2)$ for $i, j, k, l > 0$

Proof. Since these matrices and coefficients are nonnegative, the value at row r and column c of $iX_1 + jY_1$ is only zero if $X_1(r,c) = Y_1(r,c) = 0$. If that is true, then by equivalence we know $X_2(r,c) = Y_2(r,c) = 0$ and so $iX_1 + jY_1 \sim kX_2 + lY_2$.

Corollary 2.1. $A_i \sim B_i, 1 \le i \le n \implies \sum_{i=1}^n A_i \sim \sum_{i=1}^n B_i$. Again, by induction if $\sum_{i=1}^{n-1} A_i \sim \sum_{i=1}^{n-1} B_i$ then $(\sum_{i=1}^{n-1} A_i + A_n) \sim (\sum_{i=1}^{n-1} B_i + B_n)$.

Theorem 3. Let A be a set of states and $X_1 \sim X_2$ be two matrices, both with absorbing states A. Write X_1 and X_2 in canonical form [17] and calculate the mean absorption matrix of each: $B_1 = (I - Q_1)^{-1}R_1$ and $B_2 = (I - Q_2)^{-1}R_2$ (where Q_1, Q_2 are the transition probabilities between transient states and R_1, R_2

are the transition probabilities from transient states to absorbing states). Then $B_1 \sim B_2$.

Proof. $X_1 \sim X_2$ means that $Q_1 \sim Q_2$ and $R_1 \sim R_2$. Expanding $B_1 = R_1 + Q_1 R_1 + Q_1^2 R_1 + \cdots$. We see that the ith term of B_1 is $Q_1^{i-1} R_1$ and of B_2 is $Q_2^{i-1} R_2$. By Corollary 1.1, $Q_1^{i-1} R_1 \sim Q_2^{i-1} R_2$, so, by Corollary 1.2, $B_1 \sim B_2$.

Theorem 4. $X \sim Y \implies X_{abs} \sim Y_{abs}$ for any set of states made absorbing in both.

Proof. Let I be a set of indexes into the rows of X. Recall that X_{abs} is formed by setting row i of X to e_i for all $i \in I$. $X \sim Y$ means that for each row r, $X(r, \cdot) \sim Y(r, \cdot)$. Let $r_x = X_{rev}(r, \cdot)$ and $r_y = Y_{rev}(r, \cdot)$. Then either $r \in I$ and so $r_x \sim r_y$ because $r_x = r_y$ or $r \notin I$ in which case $r_x = X(r, \cdot) \sim Y(r, \cdot) = r_y$. Thus $X_{abs} \sim Y_{abs}$.

Theorem 5. $X \sim Y \implies X_{rev} \sim Y_{rev}$.

Proof. Recall that X_{rev} is formed by transposing X and then normalizing the row sums of the nonzero rows, which is equivalent to multiplying each row by the reciprocal of that row's sum (if nonzero). Each row of X_{rev} is then either the 0 vector or is a row of X^T multiplied by some nonzero constant. This means $X^T \sim X_{rev}$ and, since clearly $X \sim Y \implies X^T \sim Y^T$, by transitivity $X_{rev} \sim Y_{rev}$.

Now, let us consider CASR memory: $E_i = R_i^+ B$. In creating matrix R_i^+, rows aliased to the same observation were averaged. However, any linear combination of the rows using nonzero coefficients will produce a row equivalent to the averaged row. That is, let r be a row of R_i^+. Then r is the average of a number of vectors: $r = (e_i + e_j + \cdots + e_z)/n$, so the kth element of r is either 0 or $(1/n)$. As long as $n > 0$, $r \sim (a_1 e_i + a_2 e_j + \cdots + a_n e_z)$ when $a_j > 0$ for all j.

Similarly, we initially defined

$$N(s, s') = \frac{\sum_{a \in T_A} T_P(s, a, s')}{|T_A|}.$$

But we see

$$N(s, s') \sim \sum_{a \in T_A} \pi(s, a) T_P(s, a, s')$$

where $\pi(s, a)$ is the agent's policy: a unique probability for each state-action pair, when $\pi(s, a) > 0$ for all s and a.

We see then that any nonzero linear combination of states in making R_i^+ and any nonzero policy used in determining N results in an equivalent matrix E_i. Specifically, let $R \sim R'$ be the results of two different linear combinations of states when making R_i^+ and let $N \sim N'$ be two equivalent transition matrices derived using different policies. Then $E_i = RB \sim R'B' = E_i'$ by Theorems 1, 3, and 4.

A similar argument applies to the case of working memory. Briefly, we can see that $W_i = RN_{rev}C \sim R'N'_{rev}C = W_i'$ by Theorems 1 and 5.

Thus the seemingly arbitrary choices of averaging rows when calculating R and assuming a random policy in calculating N do not affect the ultimate results as long as some linear combination of the respective elements is used.
