# Supplementary material for: The Influence of Markov Decision Process Structure on the Possible Strategic Use of Working Memory and Episodic Memory
Source: PLoS One. 2008 Jul 23;3(7):e2756. doi: 10.1371/journal.pone.0002756 (PMC2447173; doi:10.1371/journal.pone.0002756)
Supplement: Appendix S2 — (0.66 MB PDF) [file pone.0002756.s002.pdf]

The Influence of Markov Decision Process Structure on the Possible Strategic Use of Working Memory and Episodic Memory
Appendix S2

Eric A. Zilli* and Michael E. Hasselmo

* Corresponding author. E-mail: zilli@bu.edu.

Center for Memory and Brain

2 Cummington St.

Boston University

Boston MA, 02215 USA

Appendix S2

The sequence disambiguation task consists of two sequences of states, each of which overlap at one or more observations. Figure 1 shows one simple example of this type of task. In this case, the two sequences are *red* → *green* → *magenta* and *blue* → *green* → *yellow*. The agent receives a positive reward when entering the appropriate final observation given the observation at which the current trial began. On each trial, one of the *red* or *blue* starting positions is selected randomly.

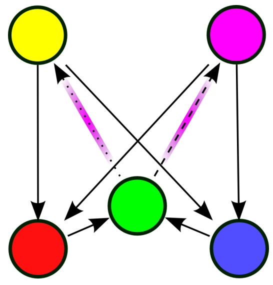

Figure 1: Aliased sequence disambiguation task. This shows the structure of the sequence disambiguation task as observed by the agent. Transition arrows colored magenta indicate transitions that may provide either positive or negative rewards (depending on where the agent started the present trial).

To further demonstrate the CASR memory analysis, we consider an AMDP where the third observation in the agent's history, instead of the second observation as in alternation, is needed to disambiguate the states at the choice point.

This task is sequence disambiguation. The task is the same as the alternation task, except for the presence of additional transitions from states $\{e_3, e_4, e_7, e_8\}$ to states $\{e_1, e_5\}$, as shown in Figure 2. The transition matrix N for this environment under a random policy is

$$N = \begin{pmatrix} 0 & 1 & 0 & 0 & 0 & 0 & 0 & 0 \\ 0 & 0 & 0.5 & 0.5 & 0 & 0 & 0 & 0 \\ 0.5 & 0 & 0 & 0 & 0.5 & 0 & 0 & 0 \\ 0.5 & 0 & 0 & 0 & 0.5 & 0 & 0 & 0 \\ 0 & 0 & 0 & 0 & 0 & 1 & 0 & 0 \\ 0 & 0 & 0 & 0 & 0 & 0 & 0.5 & 0.5 \\ 0.5 & 0 & 0 & 0 & 0.5 & 0 & 0 & 0 \\ 0.5 & 0 & 0 & 0 & 0.5 & 0 & 0 & 0 \end{pmatrix}.$$

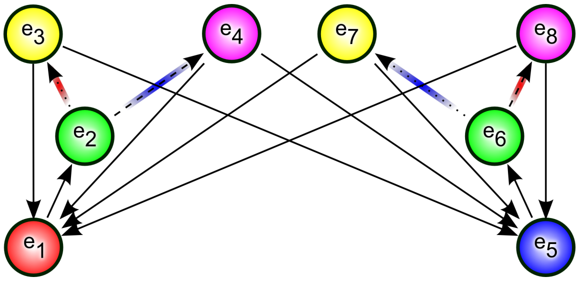

Figure 2: Underlying state-space of the sequence disambiguation task. The eight states are labeled with vectors $e_1, \ldots, e_8$. The five observations are identified by color. Solid arrows indicate transitions that result from any action. Action-specific transitions are indicated by dotted and dashed lines. Red arrows indicate transitions producing negative rewards; blue arrows indicate transitions with positive rewards. This chain differs from that of alternation only in the addition of new transitions from the top states back to the bottom states. These transitions out of the top states have equal probability of being taken..

Once again, we are concerned with the agent being able to disambiguate the *green* observation. The observation that may follow a *green* state are given by the non-zero entries in $(e_2+e_6)N$, which are $\{e_3, e_4, e_7, e_8\}$. As before, from this we find $R_1^+ = \begin{pmatrix} (e_3+e_7)/2 \\ (e_4+e_8)/2 \end{pmatrix}$. Setting the *green* states to be absorbing give us N_{abs} which we use to calculate

$$B = \begin{pmatrix} 1 & 0 \\ 1 & 0 \\ 0.5 & 0.5 \\ 0.5 & 0.5 \\ 0 & 1 \\ 0 & 1 \\ 0.5 & 0.5 \\ 0.5 & 0.5 \end{pmatrix}.$$

Finally, we find $E_1[green]$ using these two matrices:

$$
\begin{aligned}
E_1[green] &= R_1^+ B \\
&= \begin{pmatrix} (e_3 + e_7)/2 \\ (e_4 + e_8)/2 \end{pmatrix} B \\
&= \begin{pmatrix} 0.5 & 0.5 \\ 0.5 & 0.5 \end{pmatrix}.
\end{aligned}
$$

Here the rows observations are *yellow* and *magenta*, and the columns correspond to states e_2 and e_6.

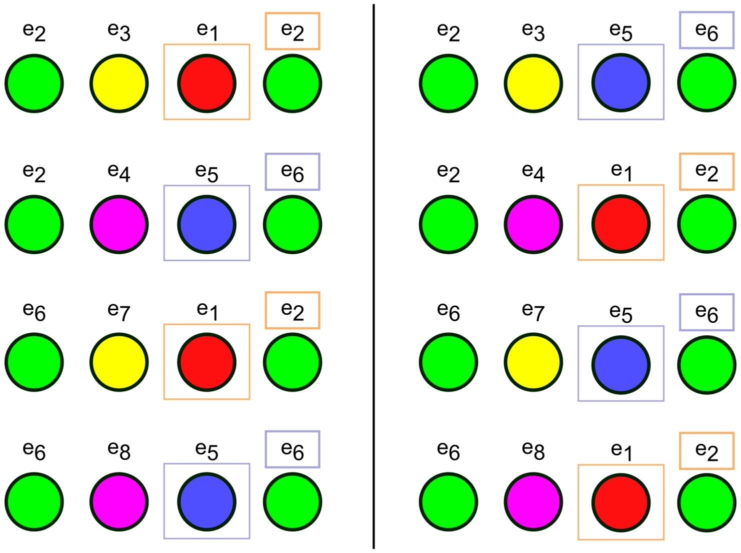

Figure 3: Possible paths/episodes from *green* to *green* in the sequence disambiguation task. The first step into the CASR memory does not predict whether the agent will be in e_2 or e_6. The second step, however, always predicts the agent's current state (end of the episode).

With all rows here equal, we find that the structure of the task results in a lack of disambiguation of *green* when using only one step of CASR memory. In simulations of this task [1], an agent using CASR memory *was* able to perform this task. The agent learned to step its CASR memory past the reward location and back to its starting location. In this simplified version of the task, this strategy corresponds to advancing CASR memory retrieval by two steps.

The rows used for the submatrix $E_1[green]$ were the nonzero entries in $(e_2 + e_6)N$. To find the submatrix $E_2[green]$, we use the nonzero entries in $(e_2 + e_6)NN_{abs}$. These are rows corresponding to states $\{e_1, e_5\}$ (notice that each is aliased to a separate observation, so no averaging of rows is done here).

$$
\begin{aligned}
E_2[green] &= R_2^+ B \\
&= \begin{pmatrix} e_1 \\ e_5 \end{pmatrix} B \\
&= \begin{pmatrix} 1 & 0 \\ 0 & 1 \end{pmatrix}.
\end{aligned}
$$

Here the rows observations are *red* and *blue*, and the columns correspond to states e_2 and e_6.

We see that by using two CASR memory retrieval advancing actions, the two *green* states are perfectly disambiguated. This is verified by examining all possible CASR memories in this task, shown in Figure 3, where the third observation, but not the second observation, predicts the final state.

Finally, the interested reader may verify the following values for 1 and 2 steps using working memory:

$$
\begin{aligned}
W_1[green] &= R_1^- N_{rand} C \\
&= \begin{pmatrix} e_1 \\ e_5 \end{pmatrix} N_{rand} \begin{pmatrix} e_2^T & e_6^T \end{pmatrix} \\
&= \begin{pmatrix} 1 & 0 \\ 0 & 1 \end{pmatrix}.
\end{aligned}
$$

$$
W_2[green] = \begin{pmatrix} 0.5 & 0.5 \\ 0.5 & 0.5 \end{pmatrix}
$$

Unlike alternation, sequence disambiguation here can only be solved by holding the observation immediate before *green* in working memory. Holding an observation that occurs two steps before *green* fails to disambiguate the *green* states.
