# Supplementary material for: The Influence of Markov Decision Process Structure on the Possible Strategic Use of Working Memory and Episodic Memory
Source: PLoS One. 2008 Jul 23;3(7):e2756. doi: 10.1371/journal.pone.0002756 (PMC2447173; doi:10.1371/journal.pone.0002756)
Supplement: Appendix S3 — (0.03 MB PDF) [file pone.0002756.s003.pdf]

The Influence of Markov Decision Process Structure on the Possible Strategic Use of Working Memory and Episodic Memory
Appendix S3

Eric A. Zilli* and Michael E. Hasselmo

* Corresponding author. E-mail: zilli@bu.edu.

Center for Memory and Brain

2 Cummington St.

Boston University

Boston MA, 02215 USA

This analysis requires a complete description of the dynamics of an environment or task. In order to extract such a description as an AMDP from a simulation of a task, we used the following simple algorithm. Essentially we perform a large number of systematic walks through the task, attempting to repeatedly sample all possible state-action transitions. This means this algorithm can only realistically extract an AMDP from a task where a finite number of random walks provide sufficient sampling of every transition.

An agent is placed into a starting state in the environment. This agent maintains a count of the number of times it has taken each action in each state. On each visit to a state, it selects the action that has been taken the fewest number of times (with ties broken randomly). On reaching a terminal state (if any exist), it is placed into a new starting state at random. For continuing tasks with non-ergodic state spaces, repeated "episodes" of a finite number of steps would have to be simulated.

While performing this systematic search of the state space, the agent maintains a count of each state-to-state transition made as well as the correspondence between states and observations (i.e. it records the aliasing function). The actual transition probabilities can then be approximated from the experienced transitions. Importantly, the transitions are stored on the basis of the true state of the underlying task, not the aliased observation (both must be made available to the agent, which is straightforward in the case of simulated tasks).

By sufficiently exploring an environment (which is only tractable for tasks with reasonably sized state spaces), the agent can have an arbitrarily good approximation of the state transitions and will know which observation occurs with each possible state. This is exactly the information needed for the analysis described in this manuscript. It should be noted, though, that this is not necessarily an efficient algorithm and it used primarily for its simplicity and because of the relatively small size of the environments used here.
